# The value of ^18^F-FDG PET/CT for the diagnosis of device-related infections in patients with a left ventricular assist device: a systematic review and meta-analysis

**DOI:** 10.1007/s00259-020-04930-8

**Published:** 2020-06-27

**Authors:** D. ten Hove, G. Treglia, R. H. J. A. Slart, K. Damman, M. Wouthuyzen-Bakker, D. F. Postma, O. Gheysens, R. J. H. Borra, G. Mecozzi, P. P. van Geel, B. Sinha, A. W. J. M. Glaudemans

**Affiliations:** 1 Department of Nuclear Medicine and Molecular Imaging, University of Groningen, University Medical Center Groningen, Hanzeplein 1, 9713GZ Groningen, The Netherlands; 2 Department of Medical Microbiology and Infection Prevention, University of Groningen, University Medical Center Groningen, Groningen, The Netherlands; 3 Clinic of Nuclear Medicine and PET/CT Centre, Imaging Institute of Southern Switzerland, Ente Ospedaliero Cantonale, Bellinzona and Lugano, Switzerland; 4 Department of Nuclear Medicine and Molecular Imaging, Lausanne University Hospital and University of Lausanne, Lausanne, Switzerland; 5 Health Technology Assessment Unit, Academic Education, Research and Innovation Area, Ente Ospedaliero Cantonale, Via Lugano 4F, CH-6500 Bellinzona, Switzerland; 6 Department of Biomedical Photonic Imaging, Faculty of Science and Technology, University of Twente, Enschede, the Netherlands; 7 University of Groningen, Department of Cardiology, University of Groningen, University Medical Center Groningen, Groningen, The Netherlands; 8 Department of Internal Medicine and infectious diseases, University of Groningen, University Medical Center Groningen, Groningen, The Netherlands; 9 Department of Nuclear Medicine, Cliniques Universitaires Saint-Luc, Brussels, Belgium; 10 Department of Radiology, University of Groningen, University Medical Center Groningen, Groningen, The Netherlands; 11 Department of Cardiothoracic Surgery, University of Groningen, University Medical Center Groningen, Groningen, The Netherlands

**Keywords:** LVAD infection, 18F-FDG PET/CT, Systematic review, Meta-analysis

## Abstract

**Background:**

Left ventricular assist devices (LVADs) are increasingly used for the treatment of advanced heart failure. LVADs improve quality of life and decrease mortality, but the driveline carries substantial risk for major infections. These device-related LVAD and driveline infections are difficult to diagnose with conventional imaging. We reviewed and analysed the current literature on the additive value of ^18^F-fluorodeoxyglucose positron emission tomography combined with computed tomography (FDG-PET/CT) imaging for the diagnosis of LVAD-related infections.”

**Materials/methods:**

We performed a systematic literature review using several databases from their inception until the 31st of December, 2019. Studies investigating the diagnostic performance of FDG-PET/CT in patients with suspected LVAD infection were retrieved. After a bias risk assessment using QUADAS-2, a study-aggregate meta-analysis was performed on a per examination-based analysis.

**Results:**

A total of 10 studies were included in the systematic review, eight of which were also eligible for study-aggregate meta-analysis. For the meta-analysis, a total of 256 FDG-PET/CT scans, examining pump/pocket and/or driveline infection, were acquired in 230 patients. Pooled sensitivity of FDG-PET/CT was 0.95 (95% confidence interval (CI) 0.89–0.97) and pooled specificity was 0.91 (95% CI 0.54–0.99) for the diagnosis of device-related infection. For pump/pocket infection, sensitivity and specificity of FDG-PET/CT were 0.97 (95%CI 0.69–1.00) and 0.93 (95%CI 0.64–0.99), respectively. For driveline infection, sensitivity and specificity were 0.96 (95%CI 0.88–0.99) and 0.99 (95%CI 0.13–1.00) respectively. Significant heterogeneity existed across studies for specificity, mostly caused by differences in scan procedures. Predefined criteria for suspicion of LVAD and/or driveline infection were lacking in all included studies.

**Conclusions:**

FDG-PET/CT is a valuable tool for assessment of device-related infection in LVAD patients, with high sensitivity and high, albeit variable, specificity. Standardization of FDG-PET/CT procedures and criteria for suspected device-related LVAD infections are needed for consistent reporting of FDG-PET/CT scans.

**Electronic supplementary material:**

The online version of this article (10.1007/s00259-020-04930-8) contains supplementary material, which is available to authorized users.

## Introduction

Left ventricular assist devices (LVADs) are an established treatment option for end-stage heart failure, either as a bridge-to-transplantation, bridge to decision or as destination therapy. LVAD treatment is associated with improvement in quality of life and improved survival. Already in 2001, LVADs have been shown to improve 1-year survival from 25 to 50% compared with conservative medical treatment [1]. With subsequent LVAD generations, outcomes have further improved, with 4-year survival for LVAD recipients now approaching that of heart transplantation (60% and 70%, respectively) [2].

However, infection of either the driveline or the LVAD pocket or pump itself still remains an important clinical problem. The overall incidence has decreased over time, but infection still occurs in 18.1% of patients during the first year after implantation and in 11.9% the years thereafter [2]. LVAD infections are associated with significant morbidity and mortality [3], in particular when complicated by bloodstream infection, which has an overall mortality rate of up to 50% [4, 5]. Establishing the diagnosis accurately and at an early stage is essential for effective management and optimal patient outcome.

The diagnosis of device-related LVAD infections mainly relies on clinical findings and results from microbiology and imaging. Swabs taken at the driveline exit site and blood cultures are a mainstay for the diagnosis, but they provide no information about the extent of an infection. Surgical removal and subsequent culture of the device is the gold standard for diagnosis, but this is often not feasible because of the severe risks associated with exchanging these devices. Imaging techniques such as echocardiography and CT angiography (CTA) are commonly used, but their diagnostic accuracy is limited due to device-related scatter artefacts, while LVAD components themselves may mimic infectious complications e.g. appearance of partially obstructed flow on echocardiography or blood between outflow graft and surrounding Gore-Tex mimicking thrombus on contrast-enhanced CT [6, 7].

Molecular imaging, and specifically ^18^F-fluordeoxyglucose positron emission tomography (FDG-PET) combined with low-dose or contrast-enhanced CT (FDG-PET/CT), is increasingly used for assessment of device-related infections. For endocarditis and infections in patients with cardiac implantable electronic devices, i.e. pacemakers, implantable cardioverter defibrillators (ICDs), FDG-PET/CT has already been incorporated in ESC guidelines and its diagnostic value for this indication is supported by an extensive body of evidence [8–11]. Its value for the diagnosis of LVAD-related infections is still being investigated, but here supporting evidence is also emerging.

The aim of this systematic review and meta-analysis is to provide a detailed overview of all evidence so far to establish the role of FDG-PET/CT in diagnosing LVAD-related infections. For the analysis, a distinction was made between driveline infections and infections of the pump/pocket.

## Research design and methods

### Screening and selection of literature

This systematic review and meta-analysis was performed according to the Cochrane methodology and PRISMA-DTA statement [12]. A comprehensive literature search was performed by two authors (DtH and GT) on PubMed, the Cochrane Library database and Embase. The search included the following terms: ‘Left Ventricular Assist Device’, ‘Infection’, ‘Driveline Infection’, ‘Endocarditis’”, and ‘Positron Emission Tomography’ or variations of these search terms. For the exact search strings, we refer to the supplemental data. Studies published up to the 31st of December 2019 were used in our analyses. Original articles that evaluated the diagnostic performance of FDG-PET/CT for suspicion of LVAD infection were eligible for inclusion in the systematic review. References in selected studies were cross-checked to find other relevant articles. Both retrospective and prospective studies as well as blinded and non-blinded studies were included. We excluded case reports and case series with small patient numbers (*n* < 5), review articles without original data, editorials, letters, and conference papers. All studies included in the systematic review were eligible for the study-aggregate meta-analysis, with exception for those with unacceptable risk of bias (e.g. no valid reference test) and/or patient overlap. Two researchers (DtH and GT) independently reviewed titles and abstracts of the retrieved articles, applying the inclusion and exclusion criteria mentioned above. The full text of the remaining articles was examined to assess their eligibility for inclusion in the study-aggregate meta-analysis. Disagreements were resolved in a consensus meeting with a third reviewer (AG).

### Data extraction and quality assessment

QUADAS-2 [13] was used to systematically assess the risk of bias and applicability concerns for all included studies. The criteria considered by QUADAS-2 are selection bias, index test bias, reference test bias, and flow-and-timing bias.

Selection bias risk was considered high if there were unexplained exclusions in the study and considered unknown when selection criteria were not (fully) described. FDG-PET/CT was considered to be the index test. Bias risk for the FDG-PET/CT scan was deemed low if the imaging specialists were blinded to the results of other diagnostic modalities and the final diagnosis of patients and if the scan was performed according to EANM/EARL procedural guidelines [14–16] These entail patient preparation with a low-carbohydrate, fat allowed diet and a period of fasting before the scan of at least 6 h and analysis of both attenuation corrected and uncorrected PET images. If assessors were not blinded and EARL/EANM procedural guidelines were not followed, the risk of bias was considered high. All studies in between, with either EARL/EANM recommendations not followed or with non-blinded assessors, were considered intermediate/unknown risk. Because the multidisciplinary consensus criteria according to the International Society of Heart and Lung transplantation (ISHLT) [17] do not constitute a true gold standard, but are currently the best known alternative, bias risk for reference test was considered intermediate for all studies that adhered to these criteria for the diagnosis. Those that deviated from ISHLT criteria were considered high risk. For flow and timing, assessment of bias risk was complicated by the fact that the ‘adequate’ time interval between index test and reference test is unknown (e.g. optimal duration of follow-up). Additionally, and in particular in the situation where patients were already treated with antibiotics at the time of FDG-PET/CT, the duration of antibiotic use may influence the value of the scan for the diagnosis, but its exact impact is unknown. Therefore, all studies were considered ‘unknown risk’ for this domain.

### Reference standard of diagnosis

For classification of the diagnosis of both driveline infections and infections of the central LVAD components (pump housing, outflow tract, and pump pocket for earlier LVAD generations, e.g. Heartmate II), we adhered to diagnostic criteria proposed in the 2011 consensus statement by the ISHLT [17] and the similar adverse event definition of device specific major infection of INTERMACS [18]. Accordingly, it was verified for all studies whether they included findings of all clinical investigations, including cultures/swabs, trans-oesophageal echocardiography, CTA if available, clinical course, and follow-up. Because of the diagnostic challenge LVAD infections may present, it was also checked whether the final diagnosis was made by a specialized multidisciplinary team, consisting of cardiologists, thoracic surgeons, infectious disease specialists, medical microbiologists, and imaging specialists, with access to all relevant clinical information in case there was any doubt about the clinical diagnosis.

### Statistical analysis

Statistical analyses were performed using Open Meta-Analyst (BROWN School of Public Health, Providence, RI, USA). Pooled subgroup analyses were performed for all included studies that evaluated FDG-PET/CT for its diagnostic value in establishing or ruling out driveline infections and/or infections of LVAD pump/pocket. Since two of the included studies only focused on FDG-PET/CT assessment of the driveline [19, 20] apart from the overall analysis of FDG-PET/CT accuracy, additional analyses were performed for driveline and central device components separately. Bivariate analysis of sensitivity and specificity was performed using likelihood ratio estimates with 95% confidence intervals (CI). An *I*^2^ higher than 50% was considered indicative of significant study heterogeneity [21]. Negative and positive likelihood ratios, as well as diagnostic odds ratios (DOR), were calculated. Negative and positive predictive values and the diagnostic accuracy were not considered as accurate since the prevalence of LVAD and/or driveline infections in the patient population of interest is unknown, violating an assumption for NPV, PPV, and diagnostic accuracy calculations. The values of negative likelihood ratio (NLR) and positive likelihood ratio (PLR) indicate to what extent the probability of having a disease decreases given a negative test result and how much the probability of the disease increases, given a positive test, respectively. The diagnostic odds ratio indicates how the probability of a correct diagnosis changes after performing the test (with a higher value indicating better performance).

## Results

### Selection of literature

**Fig. 1:**
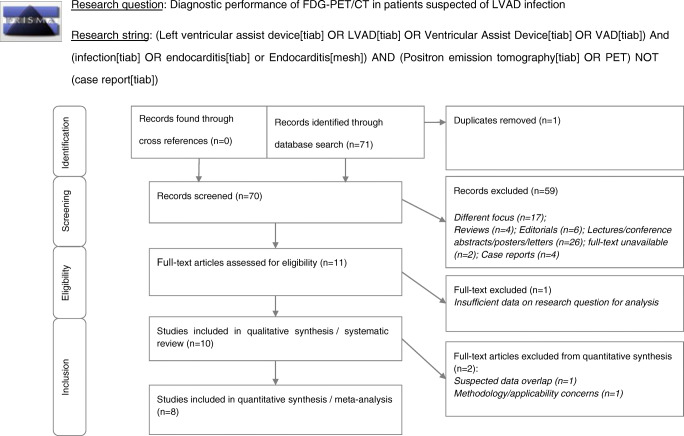
Research question: Diagnostic performance of FDG-PET/CT in patients suspected of LVAD infection Research string:(left ventricular assist device[tiab] OR LVAD[tiab] OR ventricular assist device[tiab] OR VAD[tiab]) And (infection[tiab] OR endocarditis[tiab] or endocarditis[mesh]) AND (positron emission tomography[tiab] OR PET) NOT (case report[tiab])

A total of 71 articles were identified through an electronic database search (Fig. 1). After removing one duplicate, the remaining 70 articles were screened based on title and/or abstract. Fifty-nine studies were excluded because they either had a different focus than the research question, presented no original data, or lacked a full text. Eleven studies were deemed eligible for full-text analysis. Cross-checking references for any additional publications yielded no extra results. One of the articles was excluded from further analysis because it contained insufficient data specific to our research question (only two patients in the study population had an LVAD) [22]. In total, 10 studies (*n* = 382 scans in 318 patients) were included in the systematic review. Two of the studies that were included in the systematic review were excluded from the meta-analysis: one because of suspected data overlap with a later study published by the same author [23, 24], the other because of methodology/applicability concerns based on full-text analysis [24]. The latter study included analyses of FDG-PET/CT accuracy, but microbiological diagnosis was based on driveline exit site swabs only, which cannot be used as a standalone reference test for any deeper infection of the driveline or central device components. Furthermore, this study included only patients with a relatively late stage of infection, leading to selection bias and applicability concerns. Therefore, for the meta-analysis, eight studies (*n* = 256 scans in 230 patients) were found eligible.

### Systematic review: study characteristics

In the ten articles included in the systematic review, a total of 382 FDG-PET/CT scans were acquired for 318 patients. A suspicion of LVAD-related infection was the reason for performing the FDG-PET/CT in 232 scans, 6 of which were for evaluation of treatment, while all others were considered separate episodes. The remaining 150 scans were either part of work-up for heart transplantation or assessment of pathology unrelated to LVAD (e.g. malignancy). In 78 scans, only the driveline was evaluated [19, 20]. One publication had a prospective study design [25], while all others used retrospective patient data. Median age of participants ranged from 52 to 64 years. The study population was predominantly male, proportions ranging from 77.8 to 90.5%. The characteristics of the ten included studies are summarized in Table 1.

**Table 1:** Study and patient characteristics

Authors	Year	Country	Study design	Type of patients evaluated	No. of FDG-PET/CT scans (patients)	Median age (years)	% male	Diagnosis of LVAD-specific infection^1^
Akin et al. [26]	2018	Netherlands	R	Suspected device-related infection	10 (9)	54	77.8%	8/10
Avramovic et al. [19]	2017	Germany	R	Suspected device-related infection (focus of study: driveline) or PET/CT as part of work-up for heart transplantation	48 (48)	57	83.3%	24/48
Bernhardt et al. [27]	2017	Germany	R	Suspected device-related infection	29 (21)	54	90.5%	16/29
Dell’ Aquila et al. [28]	2016	Germany	R	Suspected device-related infection	40 (31)	52	78.1%	30/40
Dell’ Aquila et al. [23]	2018	Germany	R	Suspected device-related infection	61 (47)	64	82.0%	40/61
De Vaugelade et al. [29]	2019	France	R	Suspected device-related infection	24 (22)	57	87.5%	21/24
Kanapinn et al. [20]	2019	Germany	R	Suspected device-related infection (all had baseline scan before: focus of study: driveline)	30 (30)	54	86.7%	23/30
Kim et al. [25]	2019	USA	P	Suspected device-related infection. Controls: baseline PET/CT	35 (35)	54	80.0%	28/35
Sommerlath Sohns et al. [24]	2019	Germany	R	Device-related infection, evaluation of extent of infection	85 (57)	56	86.0%	85/85
Tam et al. [30]	2019	USA	R	Suspected device-related infection	19 (18)	61	78.9%	17/19

*FDG* fluorine-18 fluorodeoxyglucose, *LVAD* left ventricular assist device, *P* prospective, *PET/CT* positron emission tomography/computed tomography, *R* retrospective

### Technical aspects

In all studies, FDG-PET scans were performed on a hybrid PET/CT system, combining an FDG-PET scan with a low-dose CT for anatomical reference and attenuation correction. In one study, the FDG-PET scan was combined with diagnostic CTA [27]. Reporting of injected activity differed between studies: while some reported an injected activity per kilogram of body weight, others reported a mean total injected activity with lower and upper ranges. The injected activity was also highly variable for included studies, ranging from 215 to 474 MBq for the mean total activity and 2.3 to 5 MBq per kg body weight (EANM guidelines advice: 2.5–5.0 MBq/kg [15]). According to study protocols, all scans were performed approximately 60 min after injection of FDG. However, the actual time intervals in clinical practice were not reported.

Visual analysis of the scans was performed in all studies; in 5 studies, this was combined with semi-quantitative analyses, using SUV_max_ [19, 20, 24] and target-to-background ratios (TBR) [23, 24, 28], reference regions being lung parenchyma and deltoid muscle [23, 28], or thoracic aorta and liver [24]. In one study, metabolic volume was also used: this was defined as the measured volume of a target lesion showing more FDG uptake than the mean FDG uptake in a delineated region of interest in the liver plus 2.5 standard deviations, with a minimum volume of 9 cm^3^ [19]. The technical details of the included studies are summarized in Table 2.

**Table 2:** Technical aspects of ^18^F-FDG PET/CT studies included in systematic review

First author, year	Imaging modality	Mean injected activity per kg and total	Time interval FDG injection and image acquisition^1^	Image analysis	Comparison to other imaging modalities
Akin et al. 2018 [26]	PET/CT(low-dose CT)	2.3 MBq/kg *μ*_tot_ = NR	60 min.	Visual analysis	None
Avramovic et al. 2017 [19]	PET/CT, (low-dose CT)	5 MBq/kg *μ*_tot_ = 338 MBq	60 min.	Visual + semi-quantitative analysis (SUV_max_ and MV)	None
Bernhardt et al. 2017 [27]	PET/CT(contrast-enhanced CT)	MBq/kg NR *μ*_tot_ = 351 MBq	60 min.	Visual analysis	None
Dell’ Aquila et al. 2016 [28]	PET/CT(low-dose CT)	5 MBq/kg *μ*_tot_ = 308 MBq	60 min.	Visual + semi-quantitative analysis (SUV_max_, SUV_mean_, TBR)	None
Dell’ Aquila et al. 2018 [23]	PET/CT(low-dose CT)	5 MBq/kg *μ*_tot_ = 344 MBq	60 min.	Visual + semi-quantitative analysis (SUV_max_, SUV_mean_, TBR)	None
De Vaugelade et al. 2019 [29]	PET/CT(low-dose CT)	3.5 MBq/kg *μ*_tot_ = 310 MBq	60 min.	Visual analysis	WBC-SPECT
Kanapinn et al. 2019 [20]	PET/CT(low-dose CT)	MBq/kg NR *μ*_tot_ = 215 MBq (1st scan) *μ*_tot_ = 218 MBq (2nd scan)	60 min.	Visual + semi-quantitative analysis (SUV_max_, SUV_mean_,)	None
Kim et al. 2019 [25]	PET/CT(low-dose CT)	MBq/kg NR *μ*_tot_ = 474 MBq	60 min.	Visual analysis	None
Sommerlath Sohns et al. 2019 [24]	PET/CT(low-dose CT)	MBq/kg NR *μ*_tot_ = NR, range 198–326 MBq	60 min.	Visual + semi-quantitative analysis (SUV_max_, TBR)	None
Tam et al. 2019 [30]	PET/CT(low-dose CT)	MBq/kg NR *μ*_tot_ = NR, range 333–370 MBq	60 min.	Visual analysis	None

*FDG* fluorine-18 fluorodeoxyglucose, *INTERMACS* Interagency Registry for Mechanical Circulatory Support, *LVAD* left ventricular assist device, *MBq* MegaBecquerel, min. minutes, μ_*tot*_ mean total injected activity, *MV* metabolic volume, *NR* not reported, *PET/CT* positron emission tomography/computed tomography, *SUV*_*max*_ maximal standardized uptake value, *SUV*_mean_ mean standardized uptake value, *TBR* target-to-background ratio, WBC-SPECT white blood cell single photon emission computed tomography

^1^According to study protocol; actual values during study not reported

### Methodological quality of included studies

**Fig. 2:**
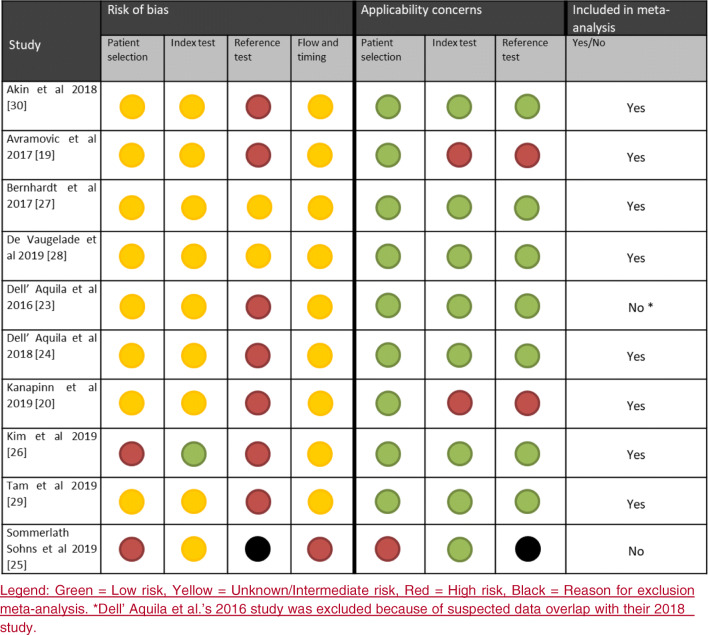
QUADAS-2 risk assessment

The QUADAS-2 risk of bias of all studies evaluated for meta-analysis eligibility is summarized in Fig. 2. Two studies had a high risk of bias for patient selection, one due to unexplained patient exclusions [25], the other because of a case series of patients with late-stage infections [24]. All other studies described a suspicion of device-related infection as inclusion criterion, but this suspicion was not further elaborated or defined. Therefore, all other studies were considered to have an unknown risk for patient selection bias. Only one study had a low risk of bias for the index test, having assessors of the FDG-PET/CT blinded to findings of other clinical tests and final diagnosis for patients, while also performing the FDG-PET/CT scan according to EANM recommendations with a high-fat, low carbohydrate diet, a pre-scan fast of more than 6 h, and assessment of both attenuation-corrected and attenuation-uncorrected images [25]. In other studies, observers were either not blinded to clinical context of patients or assessment of non-attenuation-corrected images was not described. Two studies performed the reference test fully in accordance with ISHLT recommendations [27, 29]. Two studies had high applicability concerns for both index test and reference test, because they focused on the LVAD driveline only [19, 20].

### Impact on prognosis and patient management

The ability of FDG-PET/CT to predict outcome and help inform management of device infections was discussed in three of the articles included in the systematic review [24, 25, 27]. In one study, a positive FDG-PET/CT was associated with a 50% mortality during follow-up (median survival 87.5 days), which contrasted with the non-infected group, in which no patients died during follow-up (median follow-up duration of 165 days). Twelve out of the 14 (86%) patients who died had involvement of pump or pocket infection [25]. In another study, FDG-PET/CT helped clinicians change their medical strategy for 12 out of 21 patients (57%), including four patients that were listed for high urgency heart transplantation based on FDG-PET/CT results. In all these cases, infection of the LVAD device or the deep driveline was confirmed at transplantation. [27]. In the third study, an association was found between FDG uptake of thoracic lymph nodes and adverse outcome, although this was not found for increased FDG uptake along the driveline or around any central LVAD device component [24].

### Meta-analysis: pooled diagnostic performance

In the eight articles included in the study-aggregate meta-analysis, a total of 256 FDG-PET/CT scans were acquired in 230 patients. A suspicion of device-related infection was the reason for performing FDG-PET/CT in 232 scans. In 78 scans, only the driveline was evaluated [19, 20].

For the assessment of overall device-related infections, pooled sensitivity and specificity of FDG-PET/CT were 0.95 (95% CI 0.89–0.97) and 0.91 (95% CI 0.54–0.99) respectively. NLR was 0.14 and positive likelihood ratio, PLR, was 3.54 with an overall DOR of 38.43. When only assessing the driveline, FDG-PET/CT pooled sensitivity, specificity, NLR, PLR, and DOR were respectively 0.97 (95% CI 0.88–0.99) and 0.99 (95% CI 0.13–1.0, 0.13, 3.93, and 92.46. When only assessing pump/pocket infections, FDG-PET/CT pooled sensitivity and specificity were 0.97 (95% CI 0.70–1.0) and 0.93 (95% CI 0.64–0.99) respectively. NLR was 0.12 and PLR was 5.56 with an overall DOR of 49.43.

**Fig. 3:**
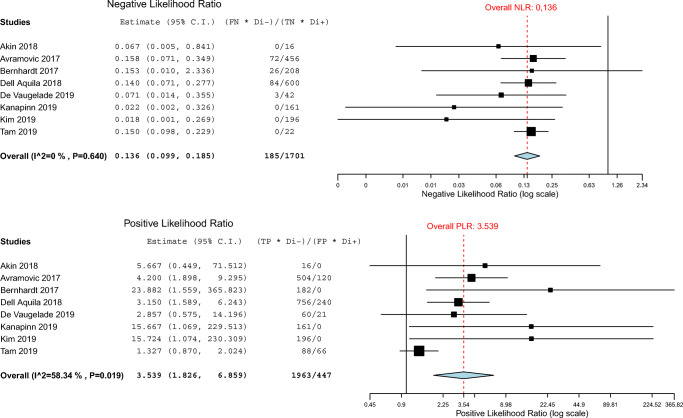
NLR and PLR forest plots for FDG-PET/CT for LVAD-specific infections (pooled analysis of driveline and LVAD)

**Fig. 4:**
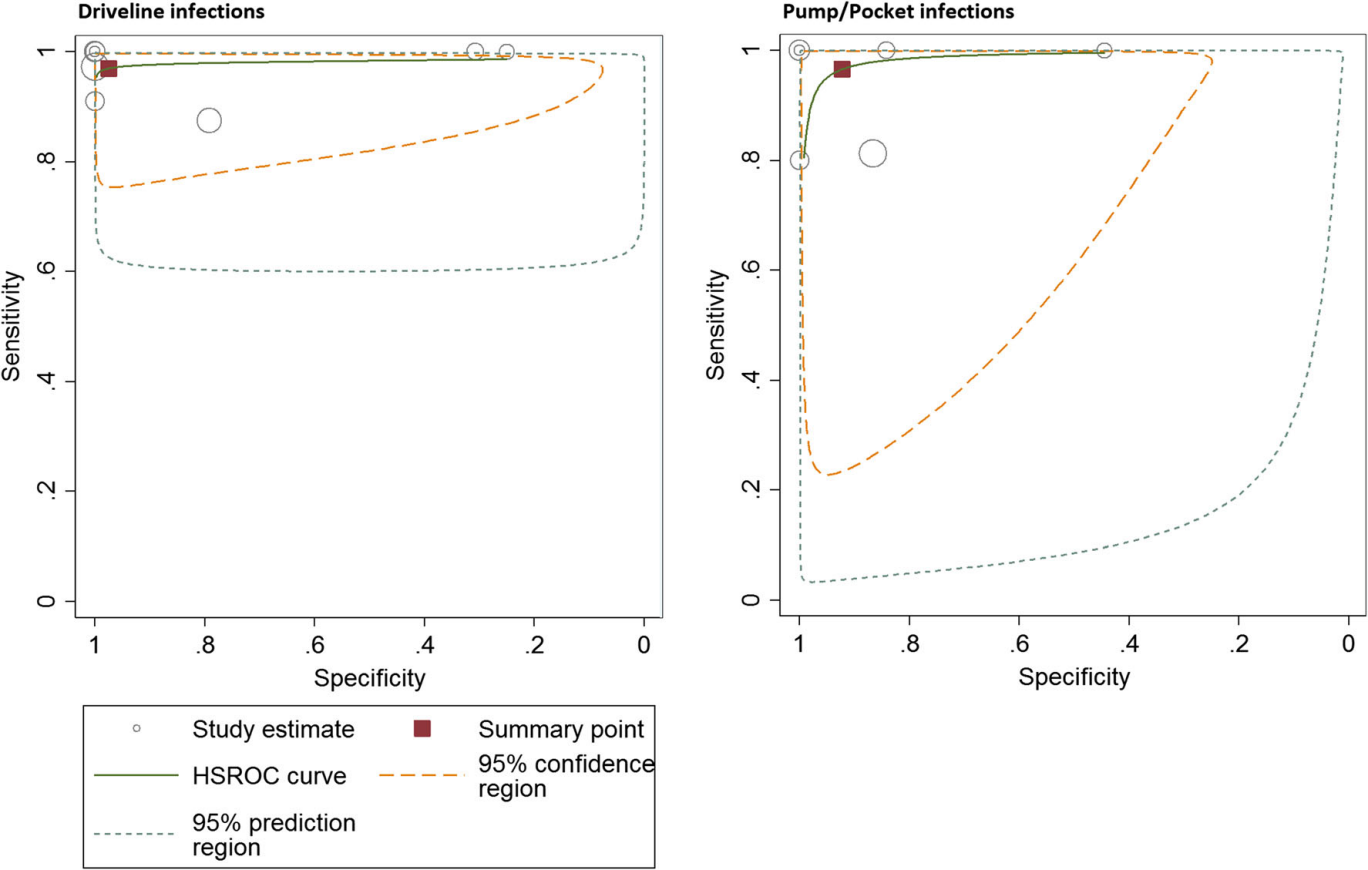
ROC curves for FDG-PET/CT for LVAD-specific infections

The *I*^2^ test for heterogeneity was positive (> 50%) for PLR of FDG-PET/CT, for assessment of driveline only, pump/pocket only, and the combination of both. Results of the meta-analysis for LVAD-specific infections, in which findings for pump/pockets and driveline are combined, are summarized in Table 3 and Fig. 3. The split analyses of driveline and pump/pocket infections are shown as ROC curves in Fig. 4. The corresponding tables and forest plots can be found under supplemental data: Tables 4 and 5, Figs. 6 and 7. Plots for FDG-PET/CT diagnostic odds ratios are represented in supplemental data Figs. 8–10.

**Table 3:** Overall diagnostic performance of FDG-PET/CT in patients with suspected LVAD and/or driveline infection

Authors, year	Reference standard for diagnostic performance assessment	True positive	False negative	False positive	True negative	Sensitivity	Specificity	PLR	NLR
Akin et al. 2018 [26]	Clinical course review by research group including medical history, comorbidities, cultures of blood and driveline (sternal wound if suspect), laboratory tests, imaging results, and outcome at end of recorded follow-up. Diagnosis according to INTERMACS definition of LVAD infection.	8	0	0	2	1.0	1.0	∞	0.00
Avramovic et al. 2017 [19]	Clinical course review at the end of recorded follow-up or transplantation: clinical evidence of infection or recurrence of symptoms, swabs at driveline exit, along driveline, surgical samples if available, and laboratory tests. Diagnosis according to INTERMACS definition of LVAD infection.	Visual 21 MV 23	3 1	5 3	19 21	0.875 0.958	0.792 0.875	4.20 7.67	0.16 0.05
Bernhardt et al. 2017 [27]	ISHLT criteria at end of follow-up, based on clinical symptoms, cultures, and swabs of exit site, along driveline and during surgery if available, and imaging data. In case of missing data, consensus diagnosis made during multidisciplinary meeting.	14	2	0	13	0.875	1.0	∞	0.12
Dell’ Aquila et al. 2016 [28]	Findings of MMB (cultures of skin and/or tissue surrounding driveline or central device components if available), surgery, clinical evidence of infection, and recurrence of symptoms at end of recorded follow-up, diagnosis according to INTERMACS definition of LVAD infection.	30	0	2	8	1.0	0.800	5.00	0.00
Dell’ Aquila et al. 2018 [23]	Clinical evidence of infection, cultures of skin and/or tissue surrounding driveline or central device components if available), surgery, and recurrence of symptoms at end of recorded follow-up. Diagnosis according to INTERMACS definition of LVAD infection.	36	4	6	15	0.900	0.714	3.15	0.14
De Vaugelade et al. 2019 [29]	ISHLT criteria at end of follow-up, based on clinical symptoms, microbiology, and imaging data. In case of missing data, consensus diagnosis made during multidisciplinary meeting.	20	1	1	2	0.952	0.667	2.86	0.07
Kanapinn et al. 2019 [20]	Consensus by 2 physicians with access to clinical criteria, findings of MMB (not further defined), and all diagnostic imaging (incl. FDG-PET/CT).	23	0	0	7	1.0	1.0	∞	0.00
Kim et al. 2019 [25]	Findings of MMB, surgery, clinical evidence of infection, and recurrence of symptoms; it was not reported who performed the reference test.	28	0	0	7	1.0	1.0	∞	0.00
Sommerlath Sohns et al. 2019 [24]	Clinician determined presence or absence of LVAD infection based on history, laboratory tests, imaging studies, and clinical outcome. Confirmation at 30 day follow-up.	11	0	6	2	1.0	0.250	1.33	0.00

*FDG* fluorine-18 fluorodeoxyglucose, *INTERMACS* Interagency Registry for Mechanical Circulatory Support, *ISHLT* International Society of Heart and Lung Transplantation, *LVAD* left ventricular assist device, *MMB* medical microbiology, *MV* metabolic volume, *PET/CT* positron emission tomography/computed tomography

While 5 studies mentioned the use of semi-quantitative analysis [19, 20, 23, 24, 28], only 3 of these described its findings in comparison with visual analysis. Visual analysis outperformed semi-quantitative analysis in 2 studies [23, 28] while in one study [19], both semi-quantitative analyses using SUV_max_ and especially metabolic volume with a cutoff of > 9 cm^3^ outperformed visual analysis, with 2/3 false negatives and 2/5 false positives correctly classified using metabolic volume.

In one study, all patients underwent two scans: a baseline scan without suspicion of infection, and a second one for assessment of driveline infection [20]. The baseline scan may have facilitated the interpretation of the second diagnostic scan, which might explain the absence of any false positives or false negatives in this study, although this warrants validation in further studies.

Analysis of false positive and false negative scans was performed in 4 studies [23, 27, 29, 30]. In one study, the cause of 2 false negatives could not be established [24]. In another, it is implied that the reason for their single false negative result might have been the 30-day period of antibiotic treatment at the time of the scan [25]. In the third study, out of 6 false positives, 4 patients had concurrent bacteraemia or other possible sources of infection, 1 patient had increased cardiac sarcoidosis activity, and 1 had a newly diagnosed chronic myeloid leukaemia. The exact effect of these comorbidities on FDG-PET/CT results in their study remained unclear. The most extensive analysis of false positives and false negatives was performed by Dell’ Aquila et al. [23]. They described prolonged antibiotic use, infection limited to the pump housing as the causes for false negatives, and the presence of chronic fistulas as main causes for a false positives in 3 cases, whereas 7 other cases remained unexplained.

Analyses of scans performed shortly after LVAD implantation showed robustness of the scan in this setting: in one study, a true negative was reported 3 weeks after LVAD implantation [26] and in another, 5 true positives and 5 true negatives were reported within 3 months after LVAD implantation [23].

## Discussion

We have pooled the data on the diagnostic value of FDG-PET/CT in detecting pump/pocket and driveline infections in patients with a LVAD to obtain more robust estimates of diagnostic performance of FDG-PET/CT in this setting. FDG-PET/CT is already included in guidelines for endocarditis and cardiovascular implantable electronic devices. Supporting evidence is emerging for the use of FDG-PET/CT in device-related infection in patients with LVADs. However, most of the reported studies have limited power, due to relatively small patient numbers enrolled and different acquisition and interpretation criteria. The separate evaluation of FDG-PET/CT accuracy for infections of LVAD pump/pocket and the driveline, next to the analysis in which these were combined, allowed us to include a significant amount of studies and patients to the analyses. In addition, we performed a further in-depth analysis of the included studies’ methodology and a stratification for driveline versus central device components, with recommendations for future studies. We performed no separate analyses for white blood cell (WBC) scintigraphy. Its diagnostic value was evaluated by only one study [29], making any pooling of data impossible beforehand. Summarily, in this study, FDG-PET/CT was found to have higher sensitivity (95.2 vs 71.9, *p* = 0.01), while a difference in specificity favouring WBC scintigraphy was not found to be statistically significant (66.7 vs 100, *p* = 0.32), although the study was underpowered to detect such a difference with only 3 negative cases. To our opinion, while potentially useful as a high specificity test in situations where FDG-PET/CT results are unclear, evidence for WBC scintigraphy so far is insufficient to make any recommendations in that regard.

### Clinical value of FDG-PET/CT in suspicion of LVAD-related infections

Considering the value of FDG-PET/CT in LVAD-related infections, we found a high overall sensitivity and specificity (both above 90%), underscoring its value in clinical practice. It was also found to have impact on prognosis and patient management. This is particularly important because of the severity of these infections and the difficulty of both their diagnosis and treatment. Accurate information about the presence and extent of an infectious process is of great importance for determining appropriate treatment e.g. duration of antibiotic treatment and/or extent of surgical debridement, while follow-up scans may be used to evaluate treatment response.

### Heterogeneity and technical considerations of FDG-PET/CT

Although the overall accuracy of FDG-PET/CT for the diagnosis of device-related infection was excellent, we also found significant heterogeneity amongst studies. The current lack of a standardized FDG-PET/CT procedure, such as the wide variety of injected activity, the possibly variable intervals between injection of the FDG and the subsequent scan, the variable use of a low carbohydrate, fat allowed diet prior to FDG-PET/CT, and missing analysis of non-attenuation-corrected PET images along with the attenuation-corrected images, may well explain the wide confidence intervals that were found for specificity of the test and the corresponding heterogeneous positive likelihood ratio. If the low carbohydrate, fat allowed diet is not used, there is a substantial risk of physiological myocardial uptake [31]. This may render any assessment of the pump housing impossible. The use of non-attenuation-corrected FDG-PET/CT images to confirm increased uptake surrounding the device is important, because the attenuation correction for the FDG-PET is based on the CT images, which means beam hardening artefacts are incorporated in the calculated FDG uptake, leading to distortions [32]. Further standardization of FDG-PET/CT protocols using the EARL criteria, applying a strict protocol for patient preparation, and providing robust interpretation criteria could substantially reduce heterogeneity caused by such confounders and increase consistency of the high overall specificity. The findings of scans performed shortly after LVAD implantation suggest that reactive inflammation after LVAD implantation may be relatively short, making FDG-PET/CT feasible early after surgery, possibly as soon as 1 month after device implantation and almost certainly 3 months after LVAD implantation.

Studies comparing visual analysis with semi-quantitative analysis found conflicting results on which of these is the most accurate during assessment of pump/pocket infections or driveline infections. Using both is probably the best approach in clinical practice until more evidence is gathered for preferring one method over the other. A clear limitation of semi-quantitative analysis is that cutoff values are not necessarily interchangeable between different PET/CT systems. This can be circumvented by calibrating the PET/CT system according to EARL or using either metabolic volume or reference regions to determine increased FDG avidity surrounding the device or driveline.

Inclusion criteria for all studies investigated were a clinical suspicion of driveline infection or infection of central LVAD components with or without a control group. However, no clearly defined criteria exist to establish a suspicion of device-related infection, which introduces a risk of selection bias e.g. overestimation of FDG-PET/CT accuracy if only performed in late-stage infection and underestimation of FDG-PET/CT accuracy if performed for incidental findings on echocardiography in spite of absent clinical signs of infection. Detailed description of the clinical presentation for all included patients can partially mitigate this risk, but to eliminate it entirely, predefined criteria for suspicion are needed.

### Proposal for structured approach in suspicion of LVAD-related infection, including FDG/PET-CT

**Fig. 5:**
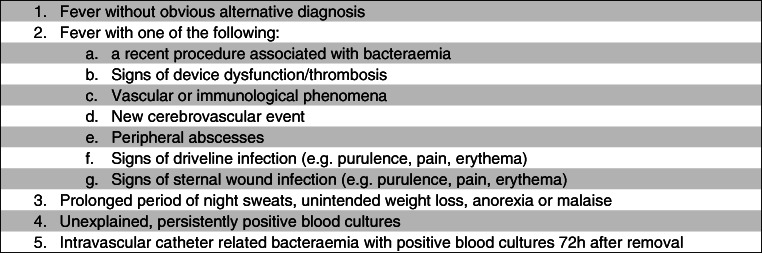
Proposed clinical signs/symptoms warranting suspicion of infection of central LVAD components

To eliminate selection bias, we propose to distinguish between pump/pocket and driveline infection, as both their assessment and clinical significance differs. Central infections would include infections of the pump pocket, and outflow tract, and, for older LVAD generations such as Heartmate II, the pump pocket. For the driveline infections, signs like localized pain and erythema with or without purulent discharge at the driveline exit site would lead to a suspicion of infection. For infection of pump or pocket, criteria could be derived from the guidelines aimed at standardizing the suspicion for infective endocarditis, as published by the BSAC [33], with adjustments for this specific patient group. Therefore, we propose the following criteria for suspecting an infection of central LVAD components; see Fig. 5. If infection of LVAD pump/pocket is suspected, FDG-PET/CT would be indicated either for establishing the diagnosis, for determining the extent of infection, or for assessing dissemination to other organs.

### Limitations

When assessing the diagnostic accuracy of any test for establishing device-specific infections in patients with an LVAD, a fundamental difficulty is the absence of a gold standard, due to associated risk of surgery and the inability of conventional investigations to accurately determine the extent of infection. Furthermore, the included studies were all relatively small and with significant differences in study protocols, leading to large heterogeneity. We took these factors into account to provide the most comprehensive review of the evidence so far.

The included studies focused almost exclusively on continuous flow LVAD systems. While this might limit the generalization of the results, these devices represent the vast majority of modern ventricular assist devices. Moreover, although the devices were almost exclusively LVADs, they were not all of the same type and/or generation, and it is certainly possible that LVADs made by different manufacturers and different materials may show different physiological uptake, impacting FDG-PET/CT accuracy. Furthermore, a difficult implantation of the device may cause a prolonged inflammatory response, impairing test accuracy, but there are currently no FDG-PET/CT data available on the impact of these factors for clinical practice.

### Conclusion

Our systematic review and meta-analysis demonstrates that FDG-PET/CT is a valuable tool for establishing or excluding the diagnosis of device specific infection in patients with a left ventricular assist device, with a high sensitivity and a high, albeit variable, specificity. Future studies, in which criteria for suspecting device infection and scan procedures are standardized, are needed to confirm that this will lead to consistently high specificity and to further elucidate the role of semi-quantitative analyses that can be used across different PET/CT systems. Despite these limitations, the current evidence strongly supports implementation of FDG-PET/CT in the standard work-up of patients with suspected LVAD-related infections, in particular when initial clinical investigations are inconclusive.

## Electronic supplementary material

ESM 1 (PDF 480 kb).

## Abbreviations

BSAC: British Society of Antimicrobial Chemotherapy
CF-LVAD: Continuous-flow left ventricular assist device
CT: Computed tomography
DOR: Diagnostic odds ratio
EANM: European Association of Nuclear Medicine
EARL: EANM Research ltd
ESC: European Society of Cardiology
FDG: 18F-fluorodeoxyglucose
ISHLT: International Society of Heart and Lung Transplantation
ICD: Implantable cardioverter defibrillator
LVAD: Left ventricular assist device
NLR: Negative likelihood ratio
NR: Not reported
PET: Positron emission tomography
PLR: Positive likelihood ratio
PRISMA-DTA: Preferred reporting items for systematic reviews and meta-analyses of diagnostic test accuracy studies
QUADAS-2: Quality Assessment of Diagnostic Accuracy Studies-version 2
SUV_max_: Maximum standardized uptake value
TBR: Target-to-background ratio
VAD: (any) ventricular assist device
