# Supplementary material for: The value of 18F-FDG PET/CT for the diagnosis of device-related infections in patients with a left ventricular assist device: a systematic review and meta-analysis
Source: Eur J Nucl Med Mol Imaging. 2020 Jun 27;48(1):241–53. doi: 10.1007/s00259-020-04930-8 (PMC7835315; doi:10.1007/s00259-020-04930-8)
Supplement: Supplementary file 1 — (PDF 480 kb). [file 259_2020_4930_MOESM1_ESM.pdf]

Supplemental Data

Includes:

Tables 4, 5

Figures 6 - 10

Search string for Embase and Pubmed

Legend for tables and figures:

FDG-PET/CT: 18F-fluorodeoxyglucose positron emission tomography / computed tomography; DOR: diagnostic odds ratio; INTERMACS: interagency registry for mechanical circulatory support; MMB = medical microbiology; MV: metabolic volume (Cut-off: $\geq$ 9cm^3) NLR: negative likelihood ratio; PLR: positive likelihood ratio.

In studies where there was insufficient data for assessment of true/false positives and negatives, these were left blank.

Table 4. Diagnostic performance of FDG PET/CT in patients with suspected infection *of **LVAD pump/pocket**.*

Authors	Reference standard for diagnostic performance assessment	True positive	False negative	False positive	True negative	Sensitivity	Specificity	PLR	NLR
Akin et al 2018	Clinical course review by research group including medical history, comorbidities, cultures of blood and driveline (sternal wound if suspect), laboratory tests, imaging results and outcome at end of recorded follow-up. Diagnosis according to INTERMACS definition of LVAD infection.	4	0	0	6	1.0	1.0	∞	0.00
Avramovic et al 2017	Clinical course review at the end of recorded follow-up or transplantation: clinical evidence of infection or recurrence of symptoms, swabs at driveline exit, along driveline, surgical samples if available and laboratory tests. Diagnosis according to INTERMACS definition of LVAD infection.	-	-	-	-	-	-	-	-
Bernhardt et al 2017	ISHLT criteria at end of follow-up, based on clinical symptoms, cultures and swabs of exit site, along driveline and during surgery if available, and imaging data. In case of missing data, consensus diagnosis made during multidisciplinary meeting.	4	1	0	13	0.800	1.0	∞	0.20
Dell' Aquila et al 2016	Findings of MMB (cultures of skin and/or tissue surrounding driveline or central device components if available), surgery, clinical evidence of infection and recurrence of symptoms at end of recorded follow up, diagnosis according to INTERMACS definition of LVAD infection	-	-	-	-	-	-	-	-
Dell' Aquila et al 2018	Clinical evidence of infection, cultures of skin and/or tissue surrounding driveline or central device components if available), surgery and recurrence of symptoms at end of recorded follow up. diagnosis according to INTERMACS definition of LVAD infection.	13	3	6	39	0.813	0.867	6.09	0.22
De Vaugelade et al 2019	ISHLT criteria at end of follow-up, based on clinical symptoms, microbiology and imaging data. In case of missing data, consensus diagnosis made during multidisciplinary meeting	5	0	3	16	1.0	0.842	6.30	0.00
Kanapinn et al 2019	Consensus by 2 physicians with access to clinical criteria, findings of MMB (not further defined) and all diagnostic imaging (incl. FDG-PET/CT)	-	-	-	-	-	-	-	-
Kim et al 2019	Findings of MMB, surgery, clinical evidence of infection and recurrence of symptoms; it was not reported who performed the reference test.	23	0	0	12	1.0	1.0	∞	0.00
Tam et al 2019	Clinician determined presence or absence of LVAD infection based on history, laboratory tests, imaging studies and clinical outcome. Confirmation at 30 day follow up.	10	0	5	4	1.0	0.444	1.80	0.00
Sommerlath Sohns et al, 2019	No reference test for extent of driveline / LVAD infection defined	-	-	-	-	-	-	-	-

Table 5. Diagnostic performance of FDG PET/CT in patients with suspected ***driveline*** infection.

Authors	Reference standard for diagnostic performance assessment	True positive	False negative	False positive	True negative	Sensitivity	Specificity	PLR	NLR
Akin et al 2018	Clinical course review by research group including medical history, comorbidities, cultures of blood and driveline (sternal wound if suspect), laboratory tests, imaging results and outcome at end of recorded follow-up. Diagnosis according to INTERMACS definition of LVAD infection.	7	0	0	3	1.0	1.0	∞	0.00
Avramovic et al 2017	Clinical course review at the end of recorded follow-up or transplantation: clinical evidence of infection or recurrence of symptoms, swabs at driveline exit, along driveline, surgical samples if available and laboratory tests. Diagnosis according to INTERMACS definition of LVAD infection.	Visual 21<br>MV 23	3<br>1	5<br>3	19<br>21	0.875<br>0.958	0.792<br>0.875	4.20<br>7.67	0.16<br>0.05
Bernhardt et al 2017	ISHLT criteria at end of follow-up, based on clinical symptoms, cultures and swabs of exit site, along driveline and during surgery if available, and imaging data. In case of missing data, consensus diagnosis made during multidisciplinary meeting.	10	1	0	18	0.910	1.0	∞	0.09
Dell' Aquila et al 2016	Findings of MMB (cultures of skin and/or tissue surrounding driveline or central device components if available), surgery, clinical evidence of infection and recurrence of symptoms at end of recorded follow up, diagnosis according to INTERMACS definition of LVAD infection	-	-	-	-	-	-	-	-
Dell' Aquila et al 2018	Clinical evidence of infection, cultures of skin and/or tissue surrounding driveline or central device components if available), surgery and recurrence of symptoms at end of recorded follow up. diagnosis according to INTERMACS definition of LVAD infection.	35	1	0	25	0.972	1.0	∞	0.03
De Vaugelade et al 2019	ISHLT criteria at end of follow-up, based on clinical symptoms, microbiology and imaging data. In case of missing data, consensus diagnosis made during multidisciplinary meeting	11	0	9	4	1.0	0.378	1.44	0.00
Kanapinn et al 2019	Consensus by 2 physicians with access to clinical criteria, findings of MMB (not further defined) and all diagnostic imaging (incl. FDG-PET/CT)	23	0	0	7	1.0	1.0	∞	0.00
Kim et al 2019	Findings of MMB, surgery, clinical evidence of infection and recurrence of symptoms; it was not reported who performed the reference test.	19	0	0	16	1.0	1.0	∞	∞
Tam et al 2019	Clinician determined presence or absence of LVAD infection based on history, laboratory tests, imaging studies and clinical outcome. Confirmation at 30 day follow up.	11	0	6	2	1.0	0.250	1.33	0.00
Sommerlath Sohns et al, 2019	No reference test for extent of driveline / LVAD infection defined	-	-	-	-	-	-	-	-

Figure 6. NLR and PLR Forest plots for FDG PET/CT for infection of LVAD pump/pocket

Negative Likelihood Ratio

Studies	Estimate (95% C.I.)	(FN * Di-)/(TN * Di+)
Akin 2018	0.108 (0.007, 1.582)	0/24
Bernhardt 2017	0.255 (0.016, 4.123)	24/120
Dell Aquila 2018	0.216 (0.099, 0.473)	135/624
De Vaugelade 2019	0.101 (0.038, 0.270)	0/80
Kim 2019	0.022 (0.001, 0.328)	0/276
Tam 2019	0.101 (0.057, 0.180)	0/40
**Overall (I^2=0 % , P=0.486)**	**0.122 (0.082, 0.183)**	**159/1164**

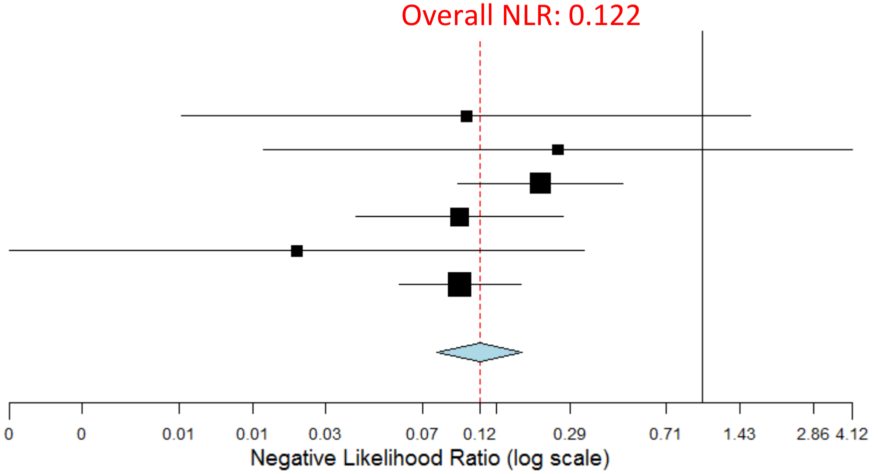

Positive Likelihood Ratio

Studies	Estimate (95% C.I.)	(TP * Di-)/(FP * Di+)
Akin 2018	12.600 (0.858, 185.047)	24/0
Bernhardt 2017	37.500 (2.320, 606.016)	96/0
Dell Aquila 2018	6.094 (2.790, 13.309)	585/96
De Vaugelade 2019	5.238 (1.963, 13.980)	95/15
Kim 2019	25.458 (1.679, 385.928)	276/0
Tam 2019	1.736 (0.976, 3.085)	90/50
**Overall (I^2=64.3 % , P=0.016)**	**5.557 (2.352, 13.130)**	**1166/161**

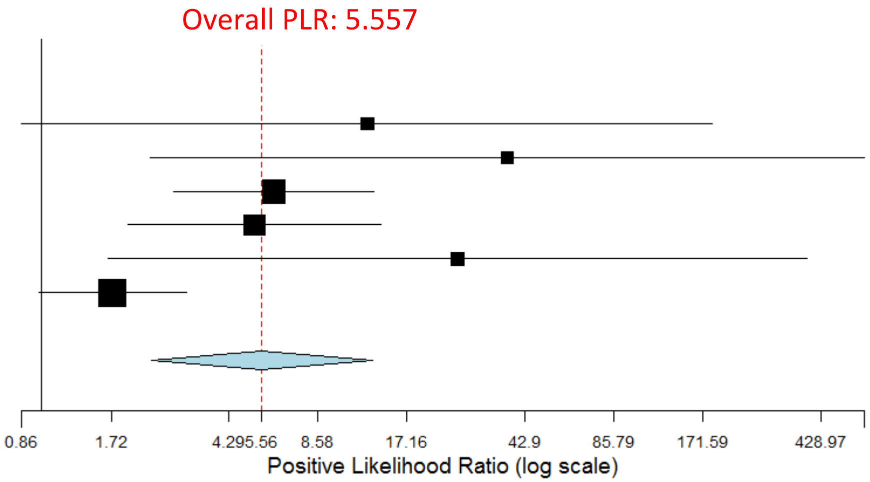

Figure 7. NLR and PLR Forest plots for FDG PET/CT for driveline infection

Negative Likelihood Ratio

Studies	Estimate (95% C.I.)	(FN * Di-)/(TN * Di+)
Akin 2018	0.071 (0.005, 0.961)	0/21
Avramovic 2017	0.158 (0.071, 0.349)	72/456
Bernhardt 2017	0.128 (0.008, 1.995)	18/198
Dell Aquila 2018	0.041 (0.003, 0.644)	25/900
De Vaugelade 2019	0.130 (0.089, 0.189)	0/44
Kanapinn 2019	0.022 (0.002, 0.326)	0/161
Kim 2019	0.026 (0.002, 0.396)	0/304
Tam 2019	0.150 (0.098, 0.229)	0/22
**Overall (I^2=0 % , P=0.718)**	**0.133 (0.103, 0.173)**	**115/2106**

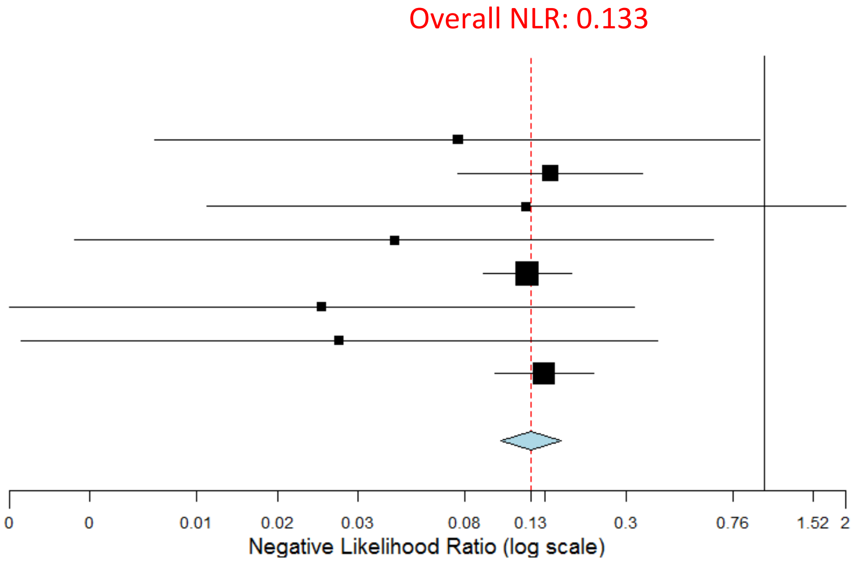

Positive Likelihood Ratio

Studies	Estimate (95% C.I.)	(TP * Di-)/(FP * Di+)
Akin 2018	7.500 (0.558, 100.873)	21/0
Avramovic 2017	4.200 (1.898, 9.295)	504/120
Bernhardt 2017	33.250 (2.140, 516.717)	180/0
Dell Aquila 2018	49.892 (3.203, 777.189)	875/0
De Vaugelade 2019	1.412 (0.966, 2.064)	143/99
Kanapinn 2019	15.667 (1.069, 229.513)	161/0
Kim 2019	33.150 (2.158, 509.121)	304/0
Tam 2019	1.327 (0.870, 2.024)	88/66
**Overall (I^2=72.98 % , P< 0.001)**	**3.934 (1.875, 8.255)**	**2276/285**

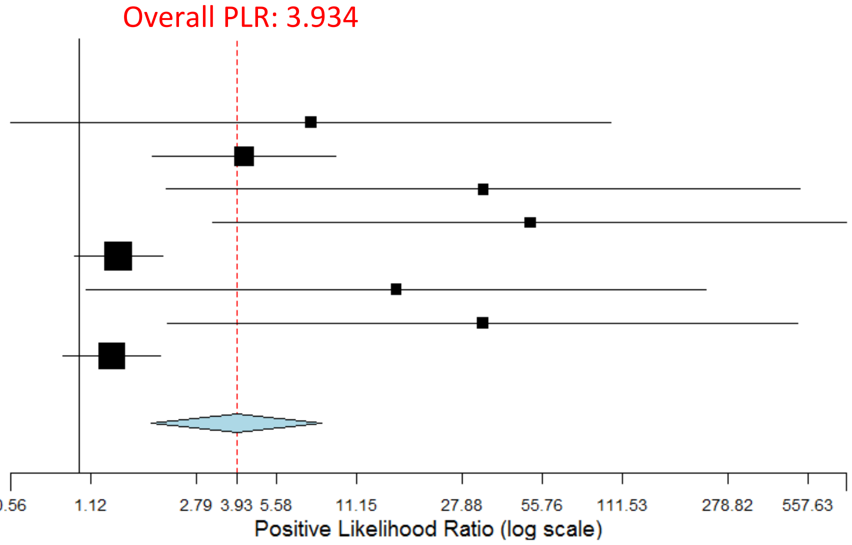

Figure 8. Forest plot Diagnostic Odds Ratio ^{18}F-FDG PET/CT for LVAD pump/pocket and driveline infection

Studies	Estimate (95% C.I.)	(TP * TN)/(FP * FN)
Akin et al 2018	85.000 (1.319, 5478.065)	16/0
Avramovic et al 2017	26.600 (5.589, 126.596)	399/15
Bernhardt et al 2017	156.600 (6.877, 3566.114)	182/0
Dell'Aquila et al - 2 2018	22.500 (5.542, 91.353)	540/24
De Vaugelade et al 2019	40.000 (1.749, 914.787)	40/1
Kanapinn et al 2019	705.000 (12.846, 38691.742)	161/0
Kim et al 2019	855.000 (15.635, 46756.257)	196/0
Tam et al 2019	8.846 (0.366, 213.798)	22/0
**Overall (I^2=0 % , P=0.445)**	**38.433 (16.535, 89.335)**	**1556/40**

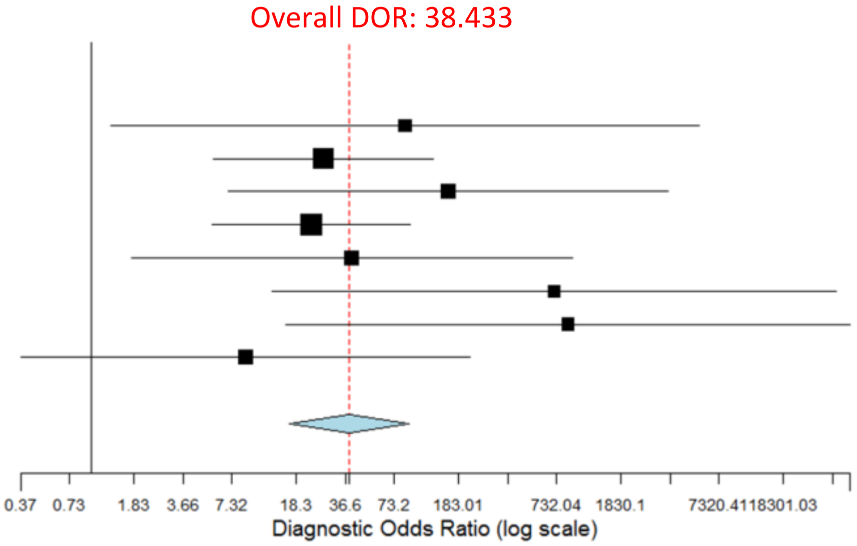

Figure 9. Forest plot Diagnostic Odds Ratio ^{18}F-FDG PET/CT for driveline infection

**Studies**	Estimate (95% C.I.)	(TP * TN)/(FP * FN)
Akin 2018	105.000 (1.705, 6464.654)	21/0
Avramovic 2017	26.600 (5.589, 126.596)	399/15
Bernhardt 2017	259.000 (9.659, 6945.214)	180/0
Dell Aquila 2018	1207.000 (47.233, 30843.682)	875/0
De Vaugelade 2019	10.895 (0.518, 229.010)	44/0
Kanapinn 2019	705.000 (12.846, 38691.742)	161/0
Kim 2019	1287.000 (24.184, 68490.014)	304/0
Tam 2019	8.846 (0.366, 213.798)	22/0
**Overall (I^2=38.96 % , P=0.119)**	**92.457 (22.711, 376.395)**	**2006/15**

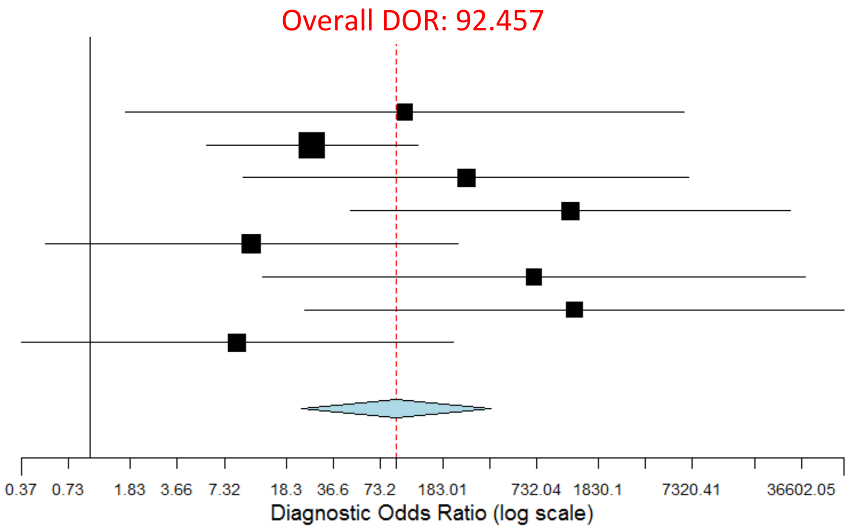

Figure 10 . Forest plot Diagnostic Odds Ratio ^{18}F-FDG PET/CT for infection of LVAD pump/pocket

Studies	Estimate (95% C.I.)	(TP * TN)/(FP * FN)
Akin 2018	117.000 (1.939, 7060.232)	24/0
Bernhardt 2017	147.000 (5.133, 4209.848)	96/0
Dell Aquila 2018	28.167 (6.152, 128.968)	507/18
De Vaugelade 2019	51.857 (2.298, 1170.117)	80/0
Kim 2019	1175.000 (21.966, 62852.646)	276/0
Tam 2019	17.182 (0.775, 380.840)	40/0
**Overall (I^2=0 % , P=0.552)**	**49.434 (16.716, 146.187)**	**1023/18**

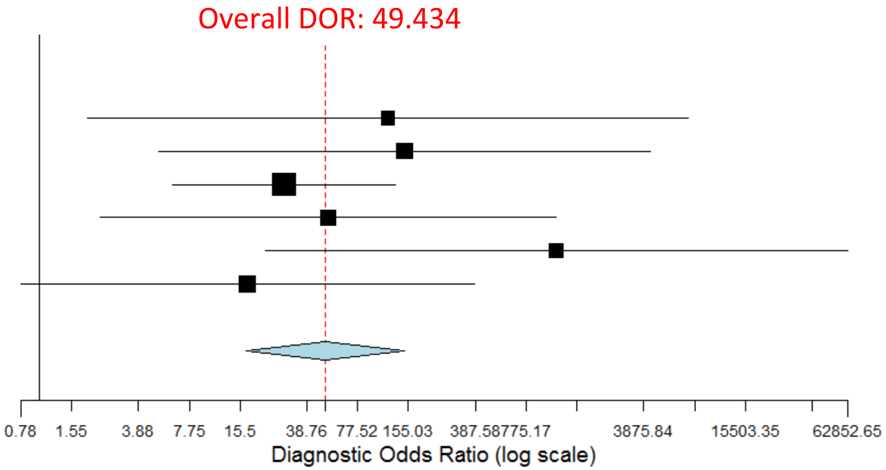

Search Strings:

Embase: ('left ventricular assist device'/exp OR 'lvad' OR 'left ventricular assist device' OR 'ventricular assist device'/exp OR 'ventricular assist device' OR 'vad' OR 'driveline infection') AND ('infection'/exp OR 'infection' OR 'bacterial endocarditis'/exp OR 'infective endocarditis') AND ('positron emission tomography'/exp OR 'positron emission tomography') NOT ('case report'/exp OR 'case report')

Pubmed: (Left ventricular assist device[tiab] OR LVAD[tiab] OR Ventricular Assist Device[tiab] OR VAD[tiab] OR Driveline infection[tiab]) And (infection[tiab] OR endocarditis[tiab] or Endocarditis[mesh]) AND (Positron emission tomography[tiab] OR PET) NOT (case report[tiab])
